# Primetime learning: collaborative and technology-enhanced studying with genuine teacher presence

**DOI:** 10.1186/s40594-018-0113-8

**Published:** 2018-05-01

**Authors:** Pekka Koskinen, Joni Lämsä, Jussi Maunuksela, Raija Hämäläinen, Jouni Viiri

**Affiliations:** 1 Nanoscience Center, Department of Physics, University of Jyväskylä, 40014 Jyväskylä, Finland; 2 Department of Education, University of Jyväskylä, 40014 Jyväskylä, Finland; 3 Department of Teacher Education, University of Jyväskylä, 40014 Jyväskylä, Finland

**Keywords:** Teacher presence, Instructional strategies, Collaborative learning, Technology-enhanced learning

## Abstract

**Background:**

Productive learning processes and good learning outcomes can be attained by applying the basic elements of active learning. The basic elements include fostering discussions and disputations, facing alternative conceptions, and focusing on conceptual understanding. However, in the face of poor course retention and high dropout rates, even learning outcomes can become of secondary importance. To address these challenges, we developed a research-based instructional strategy, the *primetime learning* model. We devised the model by organizing the basic elements of active learning into a theory-based four-step study process. The model is based on collaborative and technology-enhanced learning, on versatile formative assessment without a final exam, and on genuine teacher presence through intimate meetings between students and teachers.

**Results:**

We piloted the model in two university physics courses on thermodynamics and optics and observed persistent student activity, improved retention, and good learning outcomes. Feedback suggested that most students were satisfied with the learning experience.

**Conclusions:**

The model suits particularly well for courses that, in addition to the teaching subject itself, focus on teaching balanced study habits and strengthening social integration. By its very construction, it also helps the propagation of research-based instructional strategies. Although the model does contain challenges, it represents a generic framework for learning and teaching that is flexible for further development and applicable to many subjects and levels.

## Introduction

Science education research has been focusing on improving learning outcomes (Deslauriers et al. 2011; Freeman et al. 2014; Hake 1998). The outcomes have been measured by how well students have learned the topics under study, often reported as gains in pre- and posttests (Hake 1998). The research results recurrently urge to avoid passive lecture-type expositions (Burgan 2006) and to favor active learning methods, characterized by students actively interacting with fellow students and material at hand.

For both students and universities, however, learning outcomes are not the only relevant outcomes. They alone do not suffice. First, while teachers adopting research-based instructional strategies report higher gains in tests, too often the adoptions remain unsustainable (Henderson et al. 2012). Second, courses often suffer from poor student retention. Student activities decline as courses advance, and many students abandon courses prematurely. Fallen activities lead to a persistence problem and insinuate the gradual dropping of studying altogether (Waldrop 2015; Zwolak et al. 2017). Third, much related to the previous problem, too often of secondary importance in course designs is the individual student’s learning process and the overall learning experience. And yet, own learning experience and satisfaction is crucial for the students themselves.

Therefore, we summoned the central results from contemporary science education research and developed a new research-based instructional strategy, the *primetime learning model*. We aimed for a model that, in addition to solid learning outcomes, will improve student retention, promote research-based teaching practices, and provide a positive learning experience. In particular, we aimed for a model that is practical, requires minimal equipment and physical space, and uses valuable and limited instructional resources as efficiently as possible. The model integrates active learning components into a four-step study process, supports social integration and flexibility, and requires no final exam because it draws its power from versatile assessment. The model is transformational in its institutional novelty and assessment philosophy. In this article, in addition to introducing the model, we attempt to answer the following questions: (1) To what extent the model can improve retention and prolong activity compared with much used flipped classroom approach? (2) How well does the assessment function without an exam? (3) How do the students describe the learning experience of the model? Answering these questions helps to develop teaching models that address challenges beyond learning outcomes.

### The basic elements of active learning

According to Redish, the characteristics of active learning include student centeredness, laboratories allowing guided discoveries, explicit training for reasoning, and intellectual activities during the class (Redish 2003). Contemporary science education research provides a more detailed list of various basic elements of active learning (Table 1).

**Table 1:** The basic elements of active learning and examples for related attitudes and realizations

Basic element	Central findings
Interaction (In)	Allow students to interact with peers and teachers to articulate thoughts and arguments, challenge alternative conceptions, meet mistakes head-on and correct them (Heller et al. 1992; Herrmann 2013; Knight 2004b; Smith et al. 2009; Springer et al. 1999).
Technology enhancement (Te)	Use videos, animations, applets, simulations, and numerical exercises. Technology provides various viewpoints and controls cognitive load under well-instructed usage (De Jong and Njoo 1992; Muller et al. 2007; Schmid et al. 2014; Wagh et al. 2017; Wieman and Perkins 2005; Wieman et al. 2008).
Alternative conceptions (Al)	Do not disregard alternative conceptions, but acknowledge them and face them head-on (Beatty et al. 2006; Muller et al. 2007).
Study phenomena (Ph)	Place phenomena above abstractions and use everyday experiences to keep students on the same track; use context-rich, real-life problems (Heller and Hollabaugh 1992; Wieman and Perkins 2005).
Focus on concepts (Co)	Avoid problems with symbol manipulations and focus on concepts instead. Even math problems sprout on conceptual problems (Dufresne and Gerace 2004; Wieman et al. 2008).
Problem-solving skills (Pr)	Teach and enforce explicit problem-solving strategies (Heller et al. 1992; Heller and Hollabaugh 1992; Maloney 2011; McDermott and Redish 1999; Pedaste et al. 2015).
Self-assessment and reflection (Se)	Train metacognition by systematically promoting reflections (Beatty et al. 2006).
Feedback and formative assessment (Fo)	Give continuous and immediate feedback and build assessment that supports studying while it happens (Beatty and Gerace 2009; Dihoff et al. 2004; Dufresne and Gerace 2004; Hattie and Timperley 2007).
Multiple representations (Re)	Take advantage of context-richness, video and audio, and verbal, mathematical, and graphical representations (Heller and Hollabaugh 1992; Knight 2004a, 2004b; Treagust et al. 2017).
Adaptability (Ad)	Allow flexible and adaptable study tempo and goals and provide personal feedback (Kulik et al. 1990; Raes et al. 2014).

The categorization of the elements in the table may not be unique, but the literature does provide guidelines to tell effective learning from ineffective one. Thus, any modern learning model should be a suitable blend of these elements. The sheer knowledge of the basic elements is insufficient, however, as success or failure in teaching hinges on practical implementation and course design, as experienced both by the teachers and the students.

### The basic elements in practical course designs

The basic elements can be put into action by various research-based instructional strategies. A few of the well-established strategies in physics include Peer Instruction (Crouch and Mazur 2001; Mazur 1997), Modeling Instruction (Halloun and Hestenes 1987; Hestenes 1987), Cooperative Group Problem Solving (Heller and Heller 1999), Workshop Physics (Laws 1991), Scale-Up (Beichner et al. 2007), Just-In-Time Teaching (Novak et al. 1999), Tutorials in Introductory Physics (McDermott and Shaffer 2002), and many others. While incomplete, this list demonstrates how basic elements can be implemented at varying levels of dedication. The first level consists of course designs, where the basic elements are merely appended on top of traditional lecturing without an integrated approach to reform. While providing a low threshold to activate traditional lectures, this level is vulnerable to unsustainable adoption (Henderson et al. 2012). The second level comprises various types of flipped classroom strategies, where lectures are used for peer instruction or other student-engaging activities after the lectures’ topics have been studied at home from videos or textbooks (Crouch and Mazur 2001; Mazur 1997). These methods are much in vogue, as the course designs are still based on a safe and familiar setting of one teacher meeting the entire class in a class or an auditorium (Andrews et al. 2011). The third level of dedication blurs the distinction between lectures and recitation classes, and the students get immersed in various productive activities that happen in laboratories, studios, or computer classrooms. Related course designs are transformational compared with traditional lecturing and require more dramatic changes to teaching practices.

On large-enrollment classes, the status of the lecture is particularly prominent. Although active elements may make large lectures more engaging, the framework of one teacher and an auditorium full of students is problematic. Discussions are restricted by concerted tempo. The teacher is limited to occasional interactions with a few students, usually in the front rows. While this interaction may help the teacher to tune teaching, most students remain unheeded. Since there is not enough time available for everybody, student conversations may drift off the point, and collaborations succumb to pitfalls that make them unproductive (James and Willoughby 2011). And although brief interactions during lectures may for some students cultivate social integration, for other students they do not; it is easy for students to leave the flipped classroom without lasting social bonds, particularly for the students that otherwise prefer studying alone. In ordinary lectures, the flexibility and adaptability of student activities always remain highly restricted.

There are also other problems. Many strategies focus on restricted aspects of student activities. Some strategies have the downside of requiring dedicated, computer-equipped classrooms, whose high cost may be a hindrance (Dori et al. 2007). Often strategies focus more on student activities, less on assessment (Wieman et al. 2009). Alas, assessment dictates how students direct their study efforts in practice (Snyder 1971). Even with active learning methods, an unfavorably planned assessment can become “the silent killer of learning,” as Eric Mazur put it, and undermine teacher’s good intentions. In the literature, there are teaching methods that include several basic elements, including social integration, assessment, and multiple practices of student activation (Wells and Hestenes 1995), but we felt that there is a demand for a new method that combines the basic elements with limited institutional requirements and a high degree of practicality.

## The primetime learning model

Thus, our goal was to summon all the lessons learned from science education research and to develop a new, practical course design. We wanted the design to (i) include the basic elements of active learning to retain the good learning outcomes; (ii) be based on an assessment that improves student commitment, to promote balanced study load, and to direct students’ attention to the study process itself—to where it belongs; and (iii) support social and academic integration to reduce student drop-off (Tinto 1975).

The process of developing and refining the model took well over a year and happened within a university-wide community of 10–20 developing and practicing teachers and researchers from various branches of education research. This process enabled us to achieve both practical and theoretically sound learning model.

### Model is based on fixed groups

First, we note that many basic elements in Table 1 can be used efficiently by dividing the students into small groups. Groups provide a natural foundation for peer support (Nussbaum et al. 2009), for engaging activities, for student interactions, for facilitating formative assessment, and for implementing the course design in practice (Enghag et al. 2009; Heller et al. 1992; Springer et al. 1999). Groups are efficient vehicles to support familiarity, integration, and safe environment and to foster the feeling of belonging (Wilcox et al. 2005). These benefits even strengthen when groups are fixed and remain the same throughout the course. The relationships in the groups anchor the students into studying and help to address the persistence problem (Waldrop 2015). Most importantly, acquiring compatible friends through grouping can improve student retention (Salomone and Kling 2017) and lower drop-off rates (Wilcox et al. 2005), the very challenges we aim to address. Thus, our starting point to developing the model was to divide the students into small groups.

### Devising a new course design: arranging active learning elements into a timeline

**Fig. 1:**
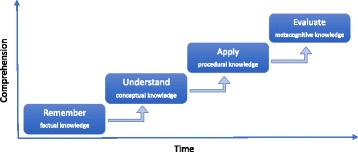
Organizing the study process into four successive sessions of increasing level of comprehension, according to Bloom’s revised taxonomy in knowledge dimension

Apart from fixed groups, we founded the new course design upon the theoretical framework of the revised Bloom’s taxonomy in knowledge dimension (Anderson and Krathwohl 2001; Krathwohl 2002). In this taxonomy, knowledge is divided into four levels: factual, conceptual, procedural, and metacognitive knowledge. Guided by this taxonomy, we arranged the study process according to four successive but temporally separate steps or sessions. The first session is about gathering and remembering the factual knowledge about a given topic. The second session is about understanding the interrelationships between the facts and about deeper, conceptual understanding. The third session is about procedural knowledge and about the skills of applying the concepts. The fourth session is about metacognitive knowledge, about self-knowledge, and about evaluating and analyzing one’s cognition. Each session can also be identified by pertinent cognitive processes (Fig. 1) (Anderson and Krathwohl 2001). These four sessions provide a solid theoretical foundation to guide the practical realization of the study process.

Then, we juxtaposed the four sessions in Fig. 1 with the basic elements of Table 1 and asked: What type of student activities the sessions should include? Which basic elements would aptly support those activities? Which activities benefit from interaction with peers? For example, since the first session focuses on factual knowledge, it should include reading and absorbing new material, which can be done alone. The relevant basic elements should then include technology enhancement (videos and simulations in technology-enhanced learning [TEL] environment), focus on phenomena and concepts (supported by material), alternative conceptions (addressed in the material), and adaptability (personal time and tempo). After considering the other three sessions the same way, we devised a timeline for the study process, with basic elements included (Table 2).

**Table 2:** Sketch for a four-step study process. Here, the elements of active learning from Table 1 are identified and assigned to the study process of Fig. 1

Knowledge dimension	Cognitive process	Active elements (from Table 1)	Example activities and notes
Factual	Remember	Te, Al, Ph, Co, Fo, Re, Ad	Expositions, books, videos. Can be done alone. The principal active element is technology enhancement.
Conceptual	Understand	In, Te, Al, Ph, Co, Se, Fo, Re, Ad	Uproot alternative conceptions and ensure correct understanding. Work through questions. The principal active element is interaction with peers.
Procedural	Apply, analyze	Pr, Te, Al, Ph, Co, Re, Ad	Problem-solving. Concepts in real life. Calculations. The principal active element is problem-solving skills.
Metacognitive	Analyze, evaluate	In, Al, Ph, Co, Se, Fo, Ad	Reflect, face mistakes, look back. The teacher has a prominent role. The principal active elements are interaction, feedback, and formative assessment.

### Practical realization of the four-step study process

**Fig. 2:**
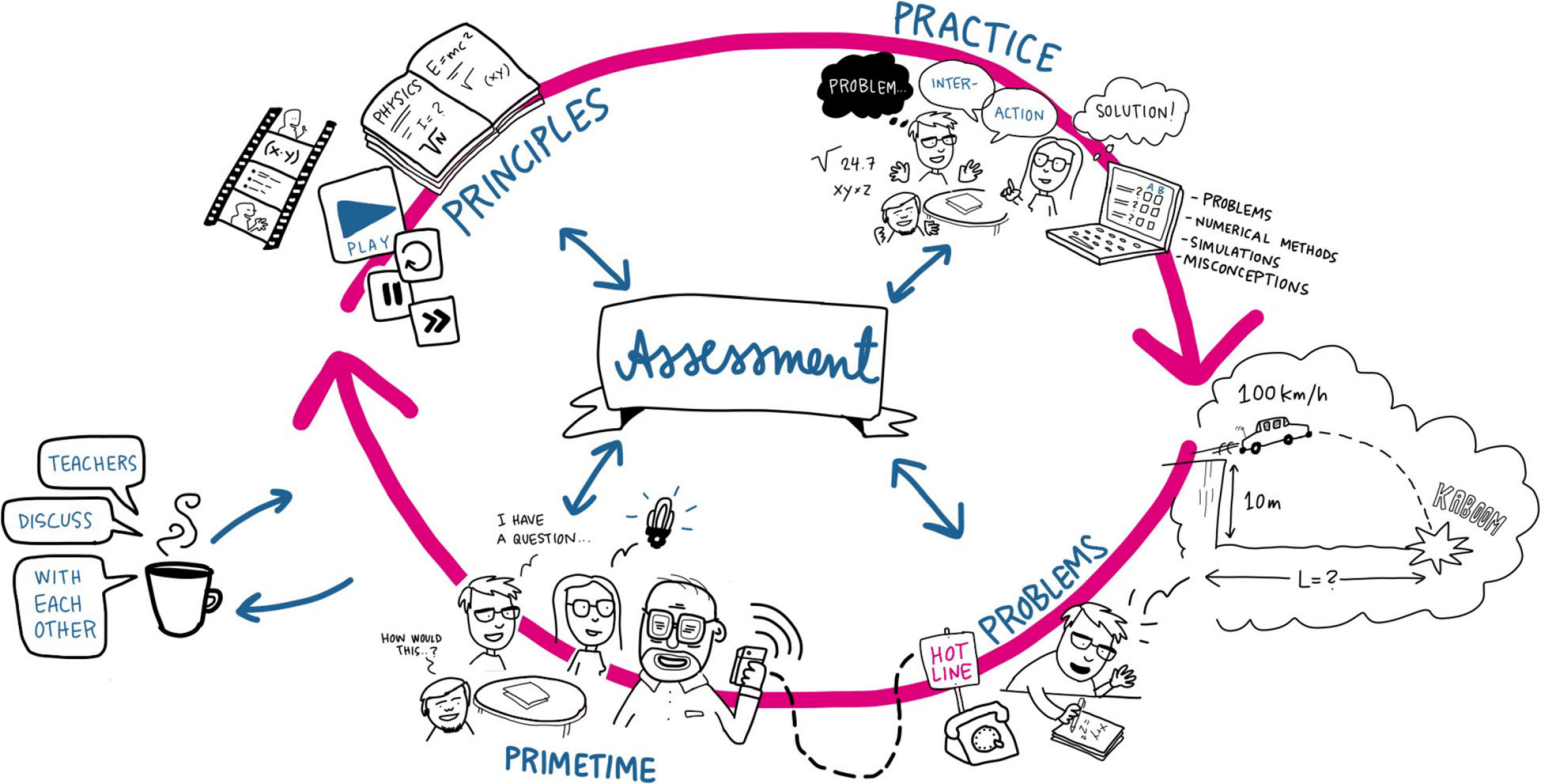
Primetime learning model with the four-step study process: principles, practice, problems, and primetime. Formative assessment underlies the entire process and motivates students to perform activities that also directly affect the grade. This drawing is a succinct summary of the primetime model (drawing courtesy of Linda Saukko-Rauta)

Now we had a solid theoretical foundation and a generic four-step process (Table 2) that we could transform into a practical realization, the primetime learning model (Table 3). For clarity, we also relabeled the four steps as (i) principles, (ii) practice, (iii) problems, and (iv) primetime (Fig. 2).

**Table 3:** The four-step study process of the primetime learning model. The process represents a practical realization of the sketch in Table 2

Step	Activity	Realization	Assessment and feedback
Principles (factual knowledge)	Study the topic alone.	Watch videos and read a book. Can be done anytime, but preferably well before the next step.	Test in TEL environment. Immediate feedback (correct answers and points).
Practice (conceptual knowledge)	Group meets to practice using the principles and concepts.	Assignments in TEL environment: conceptual questions, simulations, numerical exercises, short problems, and reflective assignments that support collaborative inquiry-based learning. Group can meet anytime and anywhere. The teacher is not present.	TEL environment offers immediate feedback (correct answers and points; group members present in the meeting share the same points).
Problems (procedural knowledge)	Apply the concepts in full-scale problem-solving.	Solve physics problems alone or collaboratively. Reinforce explicit problem-solving skills. Teacher support available when needed. Solutions (e.g., scanned papers) are submitted to TEL environment by a deadline.	After deadline, TEL environment reveals correct solutions. Students grade and correct their solutions based on given criteria. Teacher verifies corrections and gives feedback.
Primetime (metacognitive knowledge)	Students and the group receive personal support from the teacher.	Group meets teacher privately to discuss remaining problems and to reflect upon learning difficulties.	Teacher gives oral feedback for the group and each student personally.

#### Step 1. Principles: self-studying of the topic

In the first step, students use videos and a textbook to study the principles and central concepts by themselves. The emphasis is on learning the basics, on remembering the factual knowledge, and on forming an overall picture of the topic. This step is akin to the self-studying in flipped classroom (Mazur 1997). Videos give an overview, and textbooks expand the topic by examples and further details. Self-studying is assessed in the end by a test in TEL environment, which gives immediate feedback. The test aims to motivate the students to familiarize themselves with the facts and principles applied in the following steps. The instructor assembles instructional materials for study but has minimal direct interaction with students during this step.

#### Step 2. Practice: groups apply the principles

After studying the principles, the groups meet—*whenever they want*, *wherever they want*, *and without the teacher*—to put principles into practice. The emphasis is on conceptual understanding and on uprooting alternative conceptions. The group does this by completing a research-based set of assignments in the TEL environment. The assignments include visualizations, PhET and other simulations (Wieman et al. 2008), numerical problems, and context-rich, scaffolded problem-solving (Heller and Hollabaugh 1992; Kapur et al. 2008; Maloney 2011). Optimally, the assignments are open and support inquiry-based learning processes, which are known to increase both learning gains and interest in science (Pedaste et al. 2015; Raes et al. 2014). Conceptual questions, familiar from peer instruction lectures, are suitable as they are designed to address alternative conceptions and generate vivid discussions (Beatty et al. 2006).

The answers to the questions are part of the assessment and give points to group members present in the meeting, which encourages the members to collaborate and to secure answers by proper arguments (Smith et al. 2009). Small group sizes help to lower the barrier to express opinions. This organization creates positive interdependence among group members (Heller et al. 1992). After answering the assignments, the TEL environment offers correct answers and correct arguments immediately, as advised by earlier research (Dihoff et al. 2004).

The meetings are flexible, and groups can make them suit their taste. Tempo is determined by the groups’ interests, and because of the smallness of the groups, students have better chances to address individual needs. Although the teacher is absent, TEL environments can adopt some of the teacher’s routine work (Bell et al. 2010; Maloney 2011; Wagh et al. 2017). Precious contact time with teachers, as discussed later, will increase in later steps of the study process.

Principles and practices can also repeat twice before proceeding to the following steps. Such an arrangement helps to balance study load and to lessen the amount of material per session.

#### Step 3. Problems: Full-scale problem-solving

After the principles are known and rehearsed under the guidance of TEL environment, students proceed to solve full-scale problems, as familiar from traditional course designs. The emphasis is on procedural understanding, on analyzing realistic, context-rich problems, and on applying the concepts in realistic settings. Problems may be adopted from textbooks, but they should explicitly teach problem-solving skills and go beyond mere symbol manipulation. The problems can also be based on the simulations and numerical assignments used during practice sessions. For help and guidance, the teacher needs to be available for the students via a hotline. The hotline means quick, precise answers for precise questions which takes only little time from the teacher. Hotline can be arranged as scheduled availability, most easily in an online chat (Fig. 2).

Students submit personal solutions in the TEL environment by a given deadline. An easy realization is to upload scanned or photographed hand-written solutions. Model solutions are published *immediately* after the deadline (Dihoff et al. 2004). Assessment is designed such that students are made to face their mistakes by letting them check and correct their solutions, grade them based on given criteria and reupload the graded and corrected solutions in the TEL environment. In return, students get weekly feedback and brief, specific suggestions to enhance self-assessment and problem-solving skills. In other words, following research-based guidelines, students reflect upon open questions and receive immediate feedback about their successes and mistakes. The feedback is invaluable for the preparation for the next step: primetime.

#### Step 4. Primetime: quality time between group and the teacher

In the fourth and final step, the group has a *private meeting with the teacher*, the primetime meeting. By now students have already studied, practiced, and reflected on their skills so the hope is that only the most urging conceptual challenges remain to be resolved at this meeting. The subtleties of the difficult material can be worked through during a face-to-face dialog with the teacher. The step focuses on productive teacher-student interaction (Furberg 2016). This focusing enables the precious time of the teacher to be used effectively. The emphasis is therefore not on correct answers—for they are already known—but on remaining questions and challenges and on metacognitive knowledge. In other words, the teacher is at group’s disposal, and the group can take advantage of this time as it deems appropriate. For example, the group can also ask questions about following week’s new problems. The content is chosen by the group, not by the teacher. This opportunity urges the group to use the time well.

Yet the most auspicious aspect of primetime lies beyond physics, in the strong interaction and personal contact. Primetime is quality time between the group and the teacher, where quality refers to a genuine presence, meetings at an intimate level, attending to individual problems, knowing every student’s name and character, strengthening grouping, and conveying a message that the teacher cares about the students (Schoeberlein 2009). This interaction supports students’ social and academic integration, enhances their feeling of belonging, and thereby has the potential to contribute positively to student retention (Aguilar et al. 2014; Wilcox et al. 2005). Personal contact can also prevent coasting because it enables doing useful “checking” of each student. A homely atmosphere can be promoted by having primetimes in the same, informal study areas as the group meetings or, say, even at cafés around the campus area.

Primetime then completes the study process, and the students begin the same process with a new topic.

### The role of a teacher is twofold

On the one hand, thanks to personal interaction in primetime, teachers are visible mentors and coaches, real individuals that answer questions, provide guidance, and offer students genuine presence (Jennings and Greenberg 2009; Sharp and Jennings 2016). On the other hand, teachers are invisible facilitators who enable efficient studying. At the beginning of the course, they offer schedules, study environments, and opportunities for social support. During the course, they offer materials, assignments, and online help for problem-solving. Only primetimes are scheduled by teachers; other sessions are planned and run by students. Students are in charge of the study process at all times.

### Assessment powers the process

**Fig. 3:**
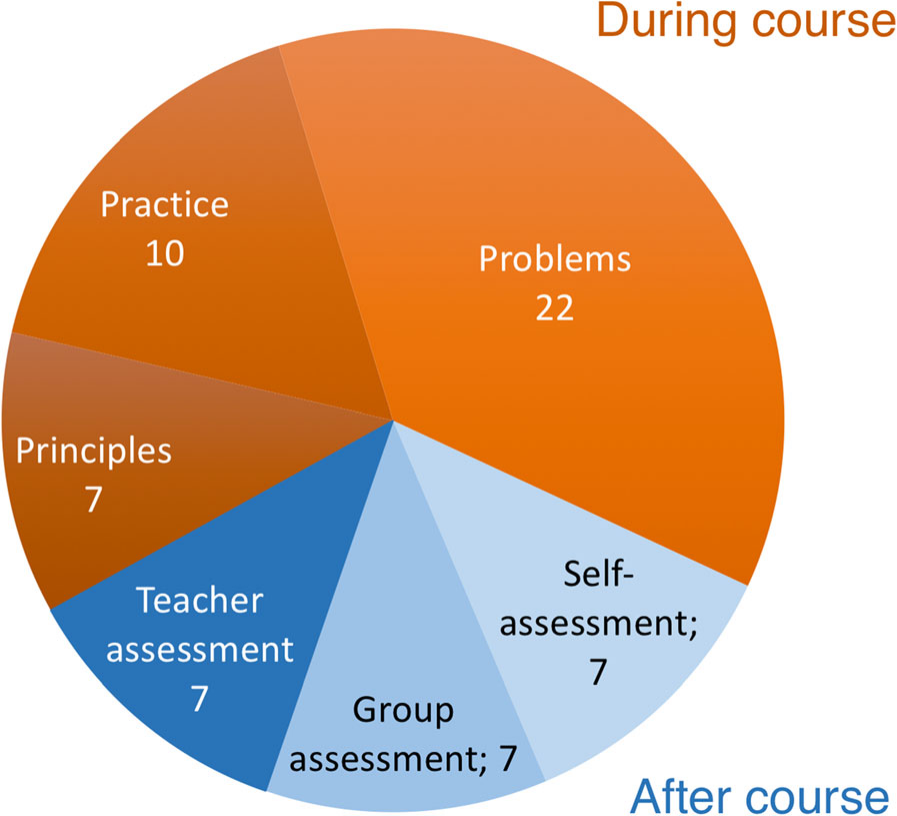
The composition of points in the grading. Points from practice, principles, and problems accumulate during the course, and points from self-, group, and teacher assessments are given at the end of the course. The maximum number of points is 60, and passing requires half of these points. The exact composition of points can be adjusted, but this is our fair estimate for a balanced compromise, where passing the course is straightforward by abiding and persistent studying and difficult by random or cherry-pick studying. Near-fail students can, if necessary, be allowed to pass the course by compensating work (Arnold 2016)

Since the process relies on independent student and group work, strategic support from the formative assessment is essential (Figs. 2 and 3). Cauley and McMillan noted that “formative assessment provides valuable information for both students and teachers” and “feedback and instructional correctives can be a powerful technique to support student motivations and achievement” (2017). Consequently, here, the purpose of assessment is not merely to grade students’ knowledge and skills, but to support and empower the study process itself to guide teachers to steer the study process and to respond to students’ difficulties (Bennett and Bennett 2017; Black et al. 2017; McManus 2008). In particular, the continuous nature of learning is best emphasized by a continuous nature of assessment (Rohrer and Pashler 2010). Because a summative exam at the end of the course would have broken these principles, it was not included in the assessment. Instead, we integrated the assessment with the model to leave minimal distinction between assessment and the study process itself.

Students accumulate points from several sources. The total number of points determines the grade and at least half of the maximum points are required for passing (Fig. 3). Most points accumulate during the course from principles, practice, and problems. Points for principles and practice come from TEL environment automatically, and points for problems come from students’ grading (verified by the teacher). Points from principles are used to motivate self-studying before group meetings, and points from practice are used to encourage students into productive group meetings. This setting supports both individual accountability and positive interdependence (Knight 2004b).

At the end of the course, the accumulated points are complemented by criteria-based self-, group, and teacher assessments. Self-assessment aims to support skills in self-reflection and metacognition (Boyd 1995; McMillan and Hearn 2008). Allowing the students’ views on own learning to influence the grade has been shown to improve motivation (McMillan and Hearn 2008). Group assessment aims to inhibit coasting and to teach cooperative learning (Joyce 1999). Each member gives the group points and verbal assessment, and all the group members share the median of individual points. The assessment criteria concern only the group and its functioning, which support the perception of positive interdependence (Johnson and Johnson 1999). Note that the majority (72%) of the points still comes from individual work and a minority (28%) from group-related work. The assessment thus represents a fair balance between individual content mastery and group activities, especially given the method’s versatile learning goals, one of which is specifically the learning of collaborative skills.

At the same time, students also give verbal peer feedback, which however is for teachers’ eyes only. Teachers analyze this feedback to ensure constructive tone, add own evaluation of student performance based on primetime meetings, and compose focused, constructive, and personal feedback for each student. At the end of the course, each student thus receives constructive verbal feedback that provides insights into course performance beyond a mere grade. Such focused interventions have been shown to trigger far-reaching positive consequences (Aguilar et al. 2014).

### Comments on student groups and the learning environment

Formal group training is useful but not necessary. While the method does put responsibility on the students and groups, the group activities are well structured and provide a safe learning environment even for the inexperienced students. After all, one of the goals of this method is the very learning of collaborative skills themselves, which takes years to learn anyway.

Optimal group composition and size are difficult questions (Harlow et al. 2016; Heller et al. 1992; Jensen and Lawson 2011), and the best choices likely depend heavily on the context. Regarding composition, Heller and Hollabaugh (1992) recommend groups of students with heterogeneous academics. We chose to group students homogenously according to how much effort they wanted to put in the course, with the hope that this homogeneity would prevent coasting. However, the conclusions on group compositions are often contradictory, so other instructors interested in using this strategy should consider grouping criteria that would best fit their contexts and institutional settings.

Choosing an appropriate group size is important for promoting productive active learning (Freeman et al. 2014). We chose a group of around four to five students so there is a diversity of ideas, but the group is small enough that every student should contribute, to minimize loafing. This size can even be considered large (Heller and Hollabaugh 1992), but it makes groups tolerate occasional and inevitable non-attendances during the course. Group size is also affected by teacher resources. By assuming that each group requires about 1 h of contact time each week, each student takes about 10–15 min of weekly contact time from one teacher. In contact teaching, teachers and teaching assistants are considered equal; for us, they are all “teachers.”

Regarding learning environment, note that the model needs no auditoriums or classrooms with specialized equipment. The only physical requirements are study areas for the group activities; the ideal environments are informal, lobby-type areas. The model only requires a suitable technology-enhanced learning (TEL) environment. The TEL environment should be able to integrate videos, simulations, queries, interactive elements, and numerical codes, preferably all in one place because full integration gives better control over the student’s workflow. The environment should be able to provide a detailed log data of student activities. Access to the TEL environment requires computers, but students’ laptops suffice well (students’ computers at home and one laptop per group in group meetings).

## Methods and materials

The primetime learning model was piloted in 2016 and 2017 on a 7-week second-year university physics course on thermodynamics and optics. The courses had 72 (2016; 25% female) and 77 (2017; 31% female) active students (both physics majors and minors) that were divided into groups of five students (14 groups in both courses). In both pilots, the groups were formed by the teacher. In 2016, groups were made homogeneous concerning the importance the students gave to the course, and in 2017, they were made based on scheduling so that each student would weekly have a maximum amount of common time available with the other group members (according to pre-course questionnaires). The courses had three teachers, consisting of one faculty member responsible for the course and two teaching assistants, one graduate and the other undergraduate student. TEL environment was The Interactive Material (TIM) (Lappalainen 2015).

The pilot courses in 2016 and 2017 are compared with the courses in 2014 and 2015. The 2014 and 2015 courses had the same content, the same teachers (except for a different undergraduate student), and a similar number of active students (97 in 2014 and 72 in 2015) with a similar gender and demographic characteristics. The only difference was the teaching method, which consisted of typical flipped classroom setting: First, self-studying from book and videos was assumed before lectures (Knight 2004a). Second, lecture time was used not for lecturing but for demonstrations and peer instruction, which consisted of students typically answering multiple-choice questions alone and after discussion with peers, following the practice made popular by Mazur (Mazur 1997). There was no lecturing. Third, lectures were followed by problem-solving and recitation classes. And fourth, the course ended with a summative exam. The maximum number of points was 60, with two points obtained from self-study tests, 12 points from problem-solving, and the remaining 46 points from an end-of-course summative exam.

To answer the first research question about student activity, we measured the number of submitted solutions in the practice phase; an equivalent measure could be used for all four courses (2014–2017). The activities and study habits of groups and individual students were analyzed using the TEL environment log data (timestamps and points for the submission of each answer in each step of the process).

To answer the second research question about assessment, we analyzed the assessment from several viewpoints. First, we compared how the distribution of the points in total varied between the years 2014 and 2017 and between different assessment criteria. Second, relating to the primetime learning model, we studied how the points from principles, practice, and problems correlated with the self-assessment points and how teacher and group assessment points are compared to self-assessment points. Third, the functioning of assessment was explored by the analysis of student’s learning outcomes using pre- and posttests on thermodynamics concepts. The pretests took place during introductory lectures and posttest a couple of weeks after finishing the section on thermodynamics. The test in 2016 was modified from an earlier study (Leinonen et al. 2013). It involved heat transfer and maximum work related to cyclic and non-cyclic processes containing isochoric, isobaric, and isothermal basic processes. The test in 2017 was Thermodynamic Concept Survey (TCS) adopted from an earlier study (Wattanakasiwich et al. 2013). It was translated from English to Finnish but otherwise given as guided by the developers. Thus, the tests in 2016 and 2017 were different, and gains are not comparable; preliminary results in this article will be systematized by further dedicated studies. The tests did not affect students’ grades but were quantified for each student using Hake’s normalized gains, defined as *g* = (*post* − *pre*)/(1 − *pre*), where the *pre* and *post* are the percentages of correct answers (Hake 1998).

To answer the third research question about the learning experience, student opinions were quantified by end-of-course questionnaires (data only from 2016). Students’ experiences about group meetings and about working without a teacher were queried by open feedback questionnaires at the end of each practice session. Student experiences were also monitored routinely by face-to-face dialogs during primetime. Experiences and possible occurrences of coasting were explored during primetime discussions and monitored by spotting anomalies in TEL log data.

## Results

### Research question 1: students followed the process rigorously

**Fig. 4:**
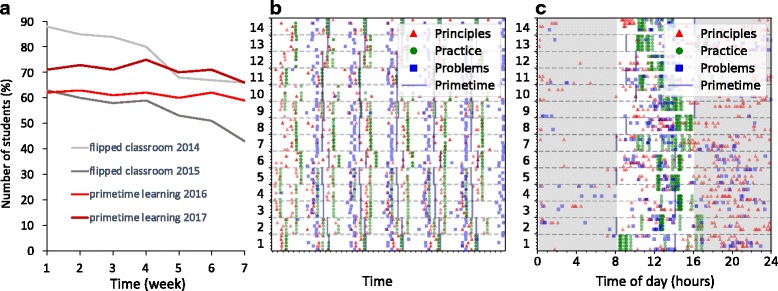
Analysis of TEL environment log data. **a** The number of active students in the course plotted using the number of students who actively solved problems. **b** Timestamps for principles (red triangles), practice (green circles), problem answer submissions (blue squares), and primetimes (vertical lines) for each student as a function of time. Groups are numbered and separated by horizontal dashed lines. Data are shown only for 2016; data for 2017 are similar. **c** The data of the panel **b** compressed to 1 day and night. Shaded areas indicate the time of day outside 8 am and 4 pm

The model was able to improve retention and prolong student activity (Fig. 4a). During earlier years, despite the particular basic elements of active learning, student activity declined considerably during the course. A common perception for the cause of this decline is that students start to wait for the exam. Here, the improved retention may have several origins: social integration, formative assessment, or the structured study process that supported balanced study habits.

The prolonged activity can also be understood in the light of study rhythm. Students acquired rapidly a steady study rhythm, which remained stable throughout the course (Fig. 4b). Principles preceded practice systematically, presumably due to encouragement from assessment. Most groups had practice during specific times, but some groups exploited their freedom to meet during more unconventional times. In overall, white regions in Fig. 4b are absent, and the distribution of symbols is similar across different groups and throughout the courses. Students thus followed the study process rigorously and well accordingly to the intended schematic of Fig. 2.

Group meetings took place mostly between 9 am and 6 pm, and practices and problem submissions took place evenly from 9 am until late midnight (Fig. 4c). Some students worked throughout the night. The deadline for the submission of problem solutions was at 2 pm on Mondays, but submissions took place also at other times. In other words, students worked whenever suitable and not just before deadlines, which helped to level the workload.

In sum, the primetime learning model indeed appeared capable of improving and prolonging student activity, at least when compared with the earlier flipped classroom approach.

### Research question 2: assessment was robust and functional

**Fig. 5:**
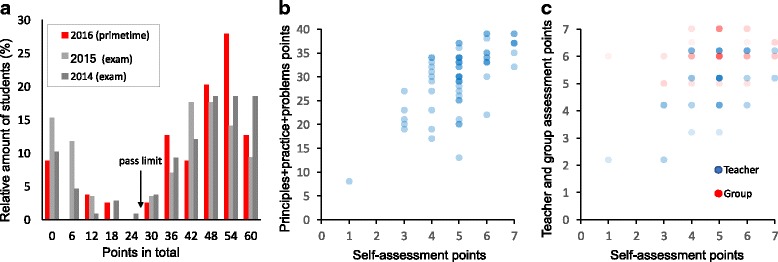
Analysis of the points in assessment. **a** The normalized distribution of total points. The pilot course is compared with two courses taught in earlier years using flipped classroom and assessed mainly by a final exam (*N*_2014_ = 108, and *N*_2015_ = 85, *N*_2016_ = 79, *N*_2017_ = 82). The pass limit is 30 points. **b** Correlation between points from self-assessment and the sum of points from principles, practice, and problems. **c** Correlation between self- and teacher assessment and self- and group assessment points. In **b** and **c**, color intensity is proportional to the frequency of occurrence. In **c**, teacher and group assessment symbols are slightly offset for clarity

The main purpose of the assessment in the primetime model was to support the study process. However, since the assessment did not contain a final exam, it still had to warrant legitimate grading and reasonable criteria for passing. Despite the lack of exams in 2016 and 2017, the total point distribution was qualitatively similar to the mainly summative assessment from previous years. However, there were two notable differences (Fig. 5a). First, the failure rate decreased. The failure rate of students with some course activity decreased from 10% (in 2014) and 15% (in 2015) down to 6% (in 2016) and 5% (in 2017). Preliminary analysis of differences in gender shows that male students benefited from this model more than female students, especially because in 2014 and 2015, the low-performing students were mostly male. Second, and most important, the majority of the failed students in the pilot course scored zero points—they had enrolled in the course but never even started studying.

Based on earlier research on different aspects of assessment (Brown et al. 1997), the assessment here seemed reliable. Students did not cherry-pick just the easy parts but were active in the entire study process. Self-assessment correlated well with the sum of points from principles, practice, and problems (*p* ≪ 0.001; Fig. 5b). The good correlation suggests a valid assessment and implies that study efforts during the course got reflected in the self-assessment. On average, the percentage of points from self-assessment was smaller than the percentage of summed points from principles, practice, and problems. Thus, if anything, students were inclined to be more self-critical than self-generous. Self-assessments correlated even with teacher assessments (Fig. 5c), despite somewhat different criteria.

Correlations between self- and group assessments show an intriguing trend: students always valued their groups high (Fig. 5c). The criteria for the group and self-assessments were different, so the assessments did not even need to correlate well. Nevertheless, it is remarkable how students valued groups high *regardless* of their own perceived performance.

**Fig. 6:**
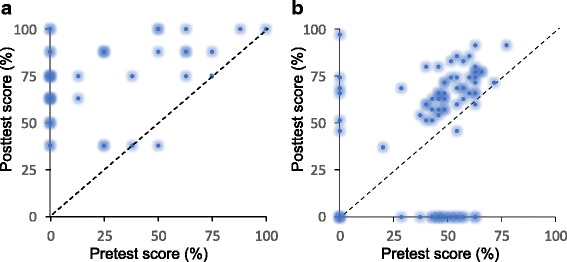
Pre- and posttest scores in **a** 2016 (*N*_pre_ = 59, *N*_post_ = 49) and **b** 2017 (*N*_pre_ = 67, *N*_post_ = 56). The intensity of each point is proportional to the number of students with the given scores. Note that the tests in **a** and **b** were different

The low failure rate and the reliability of the assessment are consistent with good learning outcomes (Fig. 6). We quantified the outcomes using Hake’s normalized gains, which were *g =* 0.63 (SD 0.33) in 2016 and *g =* 0.33 (SD = 0.20) in 2017. Although room for improvement exists, these gains represent decent learning outcomes (Hake 1998).

### Research question 3: model gave a positive learning experience

The model improved retention, leveled workload, and decreased failure rates (Figs. 4 and 5). Also, the feedback from practice sessions showed that technology-enhanced learning sessions without a teacher could surpass common interactive lectures in intensity, effectiveness, and interaction strength. In certain occasions, scaffolding by the teacher or by TEL environment would have been beneficial. However, even without teacher presence, the groups did not experience coasting as a problem.

Although only about one third of the students ended up answering the end-of-course questionnaires, the echo from the feedback had the same tone as during the course: students considered the model clear (94% agreed or agreed partly to a related claim) and functional (89%) and the workload legitimate (89%). The assessment was considered unambiguous (86%), encouraging (90%), and less stressful than exams (81%). Most of the criticism, more visible in open feedback, was related to problems in scheduling and technical issues. In particular, many students were disoriented and baffled by the lack of lectures and by the absence of an exam. Presumably, the bafflement arose mostly due to environmental factors, reflecting the deep roots that lectures and exams have in our teaching culture (Dori et al. 2007), because the feedback was otherwise positive.

Finally, students claimed the primetime model promoted new friendship (85%), appropriate responsibility of one’s studies (100%), and groups that provided a sense of belonging (100%). Thus, the model supported social integration.

In the open feedback, on the one hand, the new study routines and the lack of exam were criticized: “I learn better from standard lectures” and “…while it’s nice not to have an exam, it would be good to have ‘a real indicator’ to measure what you learned.” On the other hand, despite the new routines, most of the students considered the model valuable: “This model is a true fulfilment of peer instruction and peer discussions,” “The small group helps to realize that someone really cares and is present”, and “[Primetime teacher’s] presence and the ability to ask questions that occupy one’s mind was an excellent thing!” Students also valued learning skills beyond physics: “[The model] also taught working life skills” and “This course will be remembered just because of the group.”

## Discussion and conclusions

To clarify the institutional context, the pilot institute accepts 40–60 new physics major students, one quarter to one third of them female, practically all of them Finnish students in their early 20s. Most of these majors receive BSc in 3 years, followed by an MSc degree in an additional couple of years, with emphasis on material physics, nuclear physics, particle physics, or cosmology. The teaching language for courses at the bachelor level is Finnish, and most course participants are full-time students. The courses are usually taught by one faculty member and few teaching assistants (one assistant per 20–30 students) and the contact hours (without preparation time) are typically around 4 to 6 h per week per teacher. The instructional workload of the method was approximately on a par with the workload of more conventional teaching methods.

The model naturally has its challenges, even if cultural and institutional contexts determine their relative priority. First, some students prefer studying alone and dislike group work. However, here, the group activities are well-structured and thereby provide a secure and natural way to learn collaborative skills, which anyway should be a part of any modern science curriculum. Second, teaching assistants need to be skilled, as they alone are responsible for instructing and assessing their groups. Skills are required in both subject matter and pedagogy, especially regarding the caveats and intricacies of group dynamics (Feldon et al. 2011). Teachers must feel at ease with the spontaneity and unexpectedness of primetime meetings; after all, only the most difficult problems filter to the teacher. Teachers must be sensitive to the atmosphere in each group and, if necessary, to keep groups functional and react with timely interventions. Third, the primetime model is transformational compared with traditional course designs. Active lectures cannot be gradually migrated into the primetime model; the model requires a complete renovation of existing practices. And fourth, succesful studying requires learners to self-regulate their learning (Littlejohn et al. 2016). Zimmermann and Schunk (2012) define self-regulation as self-generated thoughts, feelings, and actions planned and cyclically adapted based on performance feedback to attain self-set goals. Self-regulated learners actively construct knowledge and use cognitive and metacognitive strategies to regulate their learning. It is argued that all learners use regulatory processes to some degree, and therefore, our future aim is to investigate self-regulation processes in the primetime learning context.

At the same time, however, the transformational nature supports the adoption of research-based instructional strategies. The model sets students in charge of their learning process, so the studying in this model—by its very construction—is less susceptible to teachers’ opinions or attitudes. After all, “active teaching” with unfit attitudes can be worse than good passive lecturing (Andrews et al. 2011).

The potential for enhanced adoption of research-based instructional strategies is also supported by teacher experiences. Both in 2016 and 2017, the teachers in the courses met weekly to share experiences and observations. All teachers, four in total, experienced primetime meetings pleasurable and empowering. Despite the large-enrollment classes, teachers learned students by name and character, and this personal contact made teaching feel meaningful and genuine. (From student’s perspective: each student knew own teacher personally.) For teachers, the pilot courses were arduous, but mainly because of novelty. In the long run, depending on class size, teacher workload ought to remain on par with flipped classroom approach—thanks to the focused contact time in the primetime meetings.

Moreover, the model comes with subsidiary benefits. It is affordable for the teacher and the institute. It can be scaled to small and large classes alike. It promotes equality by providing all the students with a similar social environment. It makes student minorities less pronounced, as studying in small groups foster a stronger sense of belonging (Aguilar et al. 2014; Madsen et al. 2013). Due to the interactive, collaborative, and structured nature of the learning process, it suits particularly well for courses that focus on teaching balanced study habits and strengthening social integration. Such courses are opportune for teaching young students group work, systematic study habits, and tools for improving metacognitive skills.

To conclude, the model is also flexible. The four steps are built on a solid theoretical foundation and therefore represent a generic framework that can easily be developed further. Consequently, our objective is to pursue the research-based development of student activities, interactions, and assignments. In particular, the primetime learning model is not specific to university physics or even other STEM subjects; it is applicable to many subjects and levels of study.

## Abbreviations

TCS: Thermodynamic Concept Survey
TEL: Technology-enhanced learning
TIM: The interactive material
